# Clinical Characteristics and Management of Headache: A Real-Life Prospective, Observational Study From a Tertiary Care Center in Eastern India

**DOI:** 10.7759/cureus.12409

**Published:** 2020-12-31

**Authors:** Soumyadarshan Nayak, Muktikanta Parida, Shanti Bhusan Das, Prabhat Kumar Padhi, Manoranjan Behera, Anant Patil, Amandeep Khurana, Santosh Kumar Swain

**Affiliations:** 1 Neurology, Srirama Chandra Bhanja Medical College and Hospital, Cuttack, IND; 2 Internal Medicine, Srirama Chandra Bhanja Medical College and Hospital, Cuttack, IND; 3 Radiodiagnosis, Srirama Chandra Bhanja Medical College and Hospital, Cuttack, IND; 4 Pharmacology, D Y Patil University - School of Medicine, Navi Mumbai, IND; 5 Internal Medicine, Khurana Clinic, Mumbai, IND

**Keywords:** clinical profile, headache, migraine, tension-type headache

## Abstract

Objective: To describe clinical profile and management pattern of headache in patients presenting to a tertiary care center.

Methods: In this observational study, demographics, radiological investigations, triggers, and treatment pattern in patients aged ≥ 14 years presenting with headache were recorded. Disability and severity of headache were assessed with Migraine Disability Assessment (MIDAS) score, Visual Analogue Scale (VAS), and Headache Impact Test (HIT-6) in case of migraineurs and VAS and HIT-6 for all other headache disorders. Patients were evaluated at baseline and after three and six months post-treatment.

Results: Out of 400 patients (60.25% females and 39.75% males), 277 (69.25%) had primary headache among whom 119 (42.96%) had migraine without aura. Stress, menstruation, fasting, and inadequate sleep were common triggers for migraine. Nausea, vomiting, photo-phonophobia and neck pain were the most common accompanying symptoms in patients with headache. Out of 106 (38.3%) patients with tension-type headache, 68.9% were episodic. In the migraine subset, 81% presented with moderate to severe disability at baseline, which changed to minimal to mild disability at three and six months post-treatment (p < 0.001). For abortive treatment, 130 (79.7%) patients were prescribed naproxen, domperidone, and sumatriptan. In 69 (42.3%) patients, valproic acid/divalproex was used for prophylaxis. Most common causes of secondary headaches (30.75%) were intracranial bleeds and cerebral venous sinus thrombosis. Most common abnormalities on computerized tomography were intracerebral hemorrhage, subarachnoid hemorrhage, sinusitis, and space-occupying lesions (SOLs).

Conclusion: In our study, migraine was the most common etiology of headache. Headache was more common in females than males, and primary headache was more common than secondary headache. Sodium valproate was the commonly used prophylaxis in migraine.

## Introduction

Headache is among the most common reason for patients to seek medical attention on a global basis, being responsible for more disability than any other neurological problem. It has been estimated that almost half of the adult population have had a headache at least once within the last year [1]. Headache disorders, which are characterized by recurrent headache, are associated with personal and societal burdens of pain, disability, damaged quality of life, and financial cost [1]. Headache classification is based on the third edition of the International Classification of Headache Disorders (ICHD-3) beta in 2013 as primary headache disorders and secondary headache disorders. The primary headache disorders include migraine, tension-type headache (TTH), trigeminal autonomic cephalalgias (TACs), and other primary headache disorders. The secondary headaches include intracranial space-occupying lesions (SOLs), infections of the central nervous system, mainly meningitis or encephalitis; subarachnoid hemorrhage; giant-cell arteritis; cerebral venous thrombosis; and idiopathic intracranial hypertension [2]. The Global Burden of Disease Study 2010 (GBD2010) reported TTH as the second most prevalent disorder worldwide and migraine as the third, but migraine far outweighs TTH as a cause of disability [3]. In a South Indian study, one-year prevalence of any headache was 63.9% with prevalence of migraine being 25.2%, and the age-standardized one-year prevalence of TTH was 35.1%. The lifetime prevalence of TTH was 52% [4]. Migraine prevalence in women exceeds than that in adult men, with female:male ratio of 2.8:1 peaking to 3.3:1 between the ages of 40 and 50 years. Female predominance is maintained in the post-menopausal age group [5]. However prior to puberty, migraine prevalence is higher in boys than in girls [6]. Limited information is available on migraine in the developing countries like India, especially in the south-eastern states [7]. Cluster headache affects up to 0.1% of the population [8]. For cluster headache, male to female ratio is approximately 2.5:1 [8]. Secondary chronic headache occurred in 2.1% of people from general population [9]. Prevalence of chronic daily headache in general population is around 4% to 5% [10]. Medication overuse headache occurs in 17%-62% of those with chronic daily headache [10].

Neuroimaging is not always necessary to confirm a distinct headache diagnosis. In order to make a responsible clinical and economically affordable decision to the patient, it is important to differentiate between a primary headache and secondary headache disorders, which are often associated with brain pathology. However, in most cases when neuroimaging is performed in headache patients, without associated neurological symptoms, the results may be negative.

The objective of the study was to describe clinical presentation, etiological profile, and management of patients presenting with headache to a tertiary care center in Eastern India.

## Materials and methods

In this prospective observational study, patients aged ≥ 14 years presenting with headache as their primary complaints were included. Demographic details and clinical features were recorded. The findings of radiological investigations in the form of computerized tomography (CT) scan [non-contrast CT (NCCT)/contrast enhanced-CT (CECT)] of brain and magnetic resonance imaging (MRI)/magnetic resonance venography (MRV) were recorded. Patients were categorized into primary and secondary headache. The etiological factors and trigger factors for headache were noted. For migraine, abortive treatment given for acute attacks and prophylaxis were recorded. For TTH, cluster and TACs treatment offered was also noted. Patients were followed up at three months and six months. Disability and severity of headache were assessed with Migraine Disability Assessment (MIDAS) score, Visual Analogue Scale (VAS) (0-10; 0: no symptoms; 10: worse symptoms), and Headache Impact Test (HIT) in case of migraineurs and VAS and HIT for all other headache disorders that were recorded at baseline, three, and six months. Correlation between MIDAS score, VAS, and HIT score at baseline versus subsequent visits was calculated. Informed written consent was taken from individual participants in local Odia language as well as in English language.

Quantitative variables are described as mean and standard deviation (SD), whereas categorical data are presented as number and percentages. Paired t-test was done between baseline and follow-up values. Pearson’s correlation test was used for correlation between HIT and MIDAS score at baseline and subsequent visits. For all statistical tests, p value < 0.05 was considered significant.

## Results

A total number of 400 patients with age ≥ 14 years presenting with chief complaint of headache were included. A total of 241 (60.25%) were females (Table 1).

**Table 1:** Baseline characteristics of study participants

Parameter	N (%)	P value
Male	159 (39.75%)	
Female	241 (60.25%)	
Age < 20 years	78 (19.5%)	<0.05
Age 21-30 years	160 (40%)	
Age 31-40 years	94 (23.5%)	
Age 41-50 years	34 (8.5%)	
Age 51-60 years	22 (5.5%)	
Age > 60 years	12 (3%)	
Outpatient (OPD)	305 (76%)	
In-patient (IPD)	95 (24%)	
Primary headache	277 (69.29%)	
Secondary headache	123 (30.75%)	

Most common age group of the headache patients in our study was 21-30 years, i.e., 160 (40%) followed by 31-40 years of age group, and the least common was from the age group above 60 years.

Primary headache was present in 277 (69.25%) patients, whereas 123 (30.75%) patients had secondary headache. In patients with primary headaches with addiction history, 83 (20.7%) were addicted to betel and 33 (8.2%) to alcohol. A total of 21 (5.2%) were smokers, and three (0.7%) were addicted to ganja (opium) (Table 2).

**Table 2:** Associated symptoms and history of addiction in patients with primary headache (n = 277)

Parameters	Results
Nausea	160 (57.76%)
Vomiting	115 (41.52%)
Photophobia/phonophobia	161 (58.12%)
Aura	44 (15.88%)
Conjunctival congestion/nasal congestion/lid edema/facial sweating	19 (6.9%)
Neck pain	109 (39.35%)
Alcohol consumption	33 (8.2%)
Pan (Betel) consumption	83 (20.7%)
Smoking	21 (5.2%)
Ganja	3 (0.7%)

Among patients with primary headache, 119 (42.96%) had migraine without aura, 44 (15.88%) had migraine with aura, and 106 (38.27%) had TTH (Table 3). Among the TTH, 73 (68.87%) patients fulfilled the criteria of episodic TTH, while 33 (31.1%) fulfilled the criteria for chronic TTH.

**Table 3:** Types of primary headache (n = 277)

Type of headache	N (%)
Migraine with aura	44 (15.88%)
Migraine without aura	119 (42.96%)
Tension-type headache	106 (38.27%)
Cluster headache	3 (1.1%)
Trigeminal autonomic cephalalgia	5 (1.8%)

Among patients with secondary headache, intracranial bleeding [intracerebral hemorrhage 18 (14.63%), subarachnoid hemorrhage 18 (14.63%), and subdural hematoma one (0.81%)] was more common followed by cerebral venous sinus thrombosis 16 (13.0%), sinusitis 14 (11.38%), and intracranial SOLs - glioblastoma multiforme four (3.25%), oligodendroma three (2.44%), astrocytoma one (0.81%), craniopharyngioma one (0.81%), ependymoma one (0.81%), and ischemic stroke [infarct four (3.25%), periventricular ischemia one (0.81%)]. Other causes of secondary headache included psychiatric disorder 13 (10.6%), idiopathic intracranial hypertension (IIH) 11 (8.9%), alcohol withdrawal three (2.44%), post-traumatic five (4.1%), medication (paracetamol) overuse three (2.44%), acute meningoencephalitis two (1.63%), hypertensive encephalopathy one (0.81%), and temporal arteritis one (0.81%).

Triggering factors in patients with migraine with aura and migraine without aura are shown in Table 4.

**Table 4:** Triggering factors for migraine

Triggering factors	Migraine with aura (n = 44)	Migraine without aura (n = 119)
Stress	25 (56.81%)	38 (31.93%)
Inadequate sleep	22 (50.0%)	34 (28.58%)
Fatigue	13 (29.6%)	19 (16.0%)
Hunger	19 (43.2%)	18 (15.1%)
Menstruation	16 (36.36%)	39 (32.78%)
Fasting	3 (6.81%)	49 (41.2%)
Non-vegetarian food	5 (11.36%)	27 (22.69%)
Dehydration	3 (6.81%)	43 (36.13%)
Bright light	9 (20.46%)	41 (34.45%)

The most common triggers for migraine without aura were fasting, dehydration, stress, inadequate sleep, and menstruation. For migraine with aura, stress, inadequate sleep, menstruation and hunger were the common triggers.

In migraine patients, 10 (6.13%) presented at baseline with grade-I (minimal disability), 20 (12.27%) presented with grade-II (mild disability), 99 (60.74%) presented with grade-III (moderate disability), whereas 34 (20.86%) presented with grade-IV (severe disability) severity of headache (Table 5).

**Table 5:** Migraine Disability Assessment (MIDAS) Score and Visual Analogue Scale (VAS) scores at baseline and follow-up visits

MIDAS score at the baseline and follow-up visits in patients with migraine			
Severity of the disease	Baseline (n = 163) N (%)	After 3 months (n = 162) N (%)	After 6 months (n = 160) N (%)
Grade 1 (0-5)	10 (6.13%)	26 (16.05%)	43 (26.88%)
Grade 2 (6-10)	20 (12.27%)	32 (19.75%)	51 (31.87%)
Grade 3 (11-20)	99 (60.74%)	104 (64.2%)	66 (41.25%)
Grade 4 (>20)	34 (20. 86%)	0	0
VAS score at the baseline and follow-up visits			
	Baseline (n = 400)	After 3 months (n = 395)	After 6 months (n = 391)
Mild (1-4)	15 (3.75%)	176 (44.55%)	235 (60.1%)
Moderate (5-7)	249 (62.25%)	205 (51.9%)	148 (37.8%)
Severe (8-10)	136 (34%)	14 (3.55%)	8 (2.1%)

For the assessment of severity at baseline, data of all patients were available; however, after three and six months, five and nine patients, respectively, were lost to follow-up.

After three months, percentages of patients presenting with grade-I, II, III, and IV severity were 16.05%, 19.75%, 64.2%, and 0%, respectively. After six months, these were 26.88%, 31.87%, 41.25%, and 0%, respectively. Follow-up of three patients after six months was not possible; one patient died after three months.

VAS score at baseline was mild in 15 (3.75%), moderate in 249 (62.25%), and severe in 136 (34%) patients. After three months, 176 (44.55%) patients were in the mild category, whereas 205 (51.9%) and 14 (3.55%) were in the moderate and severe category, respectively. After six months, 235 (60.1%) were in the mild category, whereas 148 (37.8%) and eight (2.1%) were in the moderate and severe category, respectively. Mean MIDAS score at baseline of 18.53 reduced to 14.23 after three months and 10.81 after six months. VAS score at baseline of 6.56 reduced to 4.58 after three months and 3.40 after six months. Overall 81% presented with moderate (grade 3) to severe disability (grade 4) (mean MIDAS = 18.53, VAS = 6.56) at baseline and reduced to minimal (grade 1) to mild (grade 2) disability (mean MIDAS = 14.23,VAS = 4.58) at three and six months (mean MIDAS = 10.81, VAS = 3.40), which was statistically significant (p < 0.001).

The mean HIT-6 score at onset of treatment was 51.93, which decreased to 48.59 and 46.42 after three and six months, respectively. The Pearson correlation coefficient (R) of HIT-6 score at treatment and after three months is 0.957 and after six months is 0.941, and Pearson correlation coefficient (R) between three months and six months is 0.981, which shows HIT-6 score at onset of treatment, after three months and six months are positively correlated (p value < 0.01).

For abortive treatment, 130 (79.7%) of the patients were prescribed naproxen, domperidone, and sumatriptan followed by paracetamol and ibuprofen combination in 13 (7.9%); paracetamol and tramadol combination in 12 (7.3%); and paracetamol, ergotamine, caffeine, and domperidone in combination in eight (4.9%) patients.

In 69 (42.3%) of cases, valproic acid/divalproate was used for prophylaxis followed by propranolol 23 (14.11%), flunarizine 21(12.8%), topiramate 12 (7.3%), topiramate plus amitryptiline 12 (7.3%), propranolol plus amitryptiline nine (5.5%), amitryptiline plus flunarizine two (1.2%), and only amitryptiline one (0.6%). In 14 (8.5%) of study participants, no prophylaxis was used. Prevention and lifestyle modification were advised to all (n = 163) patients, but only 57 (34.97%) followed it by three months, and 69 (42.33%) followed it by six months.

For treatment of TTH (n = 106), drugs used were amitryptiline, dexvenlafaxine, mirtazapine, escitalopram, clonazepam, and analgesics paracetamol, ibuprofen, tramadol, and combinations. For cluster headache, drugs used were divalproate, lithium, and steroids. For TACs, indomethacin, carbamazepine, and lamotrigine were used. Secondary headaches were treated according to cause.

CT scan (NCCT/CECT of brain) was performed in 329 (82.25%) cases, whereas MRI (MRI of brain/MRV/MRI of brain with cervical spine) was performed in 71 cases (17.75%). The findings of CT scan and MRI are reported in Table 6.

**Table 6:** Radiological findings in patients with headache CT: Computed tomography; CVST: cerebral venous sinus thrombosis; IIH: idiopathic intracranial hypertension; MRI: magnetic resonance imaging; MRV: magnetic resonance venography.

Finding on CT scan (n = 329)	N (%)	Finding on MRI of brain/MRV/MRI spine (n = 71)	N (%)
Astrocytoma	1 (0.30%)	CVST	16 (22.54%)
Glioblastoma multiforme	3 (0.91%)	Sinusitis	7 (9.85%)
Ependymoma	1 (0.30%)	Subarachnoid hemorrhage	4 (5.63%)
Oligodendroglioma	3 (0.91%)	Cervical spondylosis	3 (4.22%)
Sinusitis	17 (5.16%)	IIH	2 (2.81%)
Infarction	2 (0.60%)	Periventricular ischemia	1 (1.40%)
Intracerebral hemorrhage	18 (5.47%)	Infarct	2 (2.81%)
Subarachnoid hemorrhage	14 (4.25%)	Meningoencephalitis	2 (2.81%)
Subdural hematoma	1 (0.30%)	Craniopharyngioma	1 (1.40%)
Normal	269 (81.76%)	Glioblastoma multiforme	1 (1.40%)
		Normal	22 (30.98%)

Most common pathologic findings on CT brain were intracerebral hemorrhage 18 (5.47%) followed by sinusitis 17 (5.16%) and subarachnoid hemorrhage (SAH) 14 (4.25%). Out of the 71 MRI, the most common finding was cerebral venous sinus thrombosis (CVST) 16 (22.54%) followed by sinusitis seven (9.85%), SAH four (5.63%), cervical spondylosis three (4.22%), infarct two (2.81%), meningoencephalitis two (2.81%), IIH two (2.81%), periventricular ischemia one (1.40%), craniopharyngioma one (1.40%), and glioblastoma multiforme one (1.40%). Other 30.98% cases were normal.

## Discussion

In this prospective observational study, we evaluated clinical presentation, investigations, and management pattern of patients presenting with headache at the tertiary care center. In our study, 40% patients were from the age group of 21-30 years. Our observations are similar to other studies reporting maximum number of patients from this age group [11]. In our population, the female to male ratio was approximately 3:2, suggesting higher rates of headache in females as compared to males. Other studies have reported even more incidence rates in female patients (7.4:2.6 and 6.2:3.8, respectively) [11,12]. Influence of hormones plays an important role in primary headache in females [13]. Primary headache was more common than secondary headache in our study. This observation is also in similar lines with published literature [11,14]. Among the patients with primary headache, migraine (with and without aura) was the most common type of headache, in our study followed by TTH. Another study reported TTH as the most common type of headache [14]. Among secondary headaches, intracranial bleeding was the most common cause followed by CVST, sinusitis, and intracranial SOLs. A total of 25% of our headache patients required hospitalization. Owolabi et al. had reported migraine with aura in 42% and without aura in 58% [10]. In our study, 71.2% of patients were migraine without aura. The common triggering factors for migraine with/without aura were fasting, stress, menstruation, inadequate sleep, and hunger. Occurrence of migraine may be influenced by menstruation, pregnancy, and hormonal therapies in females [15]. Nausea, vomiting, photo-phonophobia, and neck pain were the most common accompanying symptoms in headache patients in our study. Most common addiction history in our study participants was pan (betel) addiction. 

TTH, the most common type of primary headache worldwide, can be episodic or chronic [16]. A total of 38.3% patients fulfilled criteria for tension-type headache in our study of whom 68.9% were categorized as episodic and 31.1% as chronic TTH. In another study from Southeast Asia, the incidence of TTH ranged from 20% to 40% with a female preponderance [17]. The incidence of cluster and TACs in our study was low, probably because of duration of study.

In our study, in the migraine subset, 81% presented with moderate (grade 3) to severe disability (grade 4) at baseline and significantly reduced to minimal (grade 1) to mild (grade 2) disability at three and six months. A strong positive correlation was observed with reduction in the HIT-6 score at three and six months from baseline. Correlation was observed with increasing duration of therapy and primary preventive measures.

Use of non-steroidal anti-inflammatory drugs and triptans is common in the treatment of acute attack of migraine. Anti-emetics are also commonly used for treatment of nausea and vomiting. In our study for abortive treatment, maximum number of patients received naproxen, domperidone, and sumatriptan. This was followed by paracetamol and ibuprofen combination; paracetamol and tramadol combination; and paracetamol, ergotamine, caffeine, and domperidone in combination. Review of literature suggests higher incidence of all-adverse events with sumatriptan than diclofenac-potassium and ibuprofen [18]. In our study group, 42.3% patients received valproic acid/divalproex sodium for prophylaxis. It should be very careful in female patients with child-bearing potential [19].

For treatment of TTH, drugs most commonly used were tricyclic antidepressants, analgesics, muscle relaxants, and combinations. The mean HIT-6 and VAS scores overall showed a significant improvement at three and six months post-treatment. Among patients with secondary headache, intracranial bleeding followed by CVST and intracranial SOLs were the most common. Most of the patients in our study had normal CT scan findings (81.8%) as reported in another study [15]. However, MRI was abnormal in 69% cases. Most common abnormalities on CT were intracerebral hemorrhage, SAH, sinusitis, and SOLs, while most common abnormalities detected on MRI were CVST, sinusitis, and SAH.

Overall, our study provides significant insights in terms of presentation of patients with headache and its management pattern. Ours was a single center study. Larger multicenter studies are required to confirm our observations.

## Conclusions

In our study population, migraine was the most common etiology of headache, followed by TTH. Identification of secondary headaches is important and warrants active management. Detailed clinical history is necessary for the patients presenting with headache as their primary complaints, and timely radiological investigations are necessary for diagnosis of the etiology of the headache. Severity disability in primary headaches, especially migraine, can be prevented by proper prophylaxis. The study showed higher rates of headache in females than male patients and primary headache being more common than secondary headache. Sodium valproate is commonly used prophylaxis in migraine.

## References

[1] (2020). Headache disorders. https://www.who.int/news-room/fact-sheets/detail/headache-disorders.

[2] (2018). Headache classification committee of the international headache society (IHS). The international classification of headache disorders, 3rd edition. Cephalalgia.

[3] Stovner Lj, Hagen K, Jensen R (2007). The global burden of headache: a documentation of headache prevalence and disability worldwide. Cephalalgia.

[4] Abu-Arafeh I, Razak S, Sivaraman B, Graham C (2010). Prevalence of headache and migraine in children and adolescents: a systematic review of population-based studies. Dev Med Child Neurol.

[5] Stewart WF, Lipton RB, Celentano DD, Reed MI (1992). Prevalence of migraine headache in the United States. Relation to age, income, race and other socio demographic factors. JAMA.

[6] Mortimer MJ, Kay J, Jaron A (1992). Epidemiology of headache and childhood migraine in an urban general practice using ad hoc, Valquist and HIS criteria. Dev Med Child Neurol.

[7] Wei DYT, Ong JJY, Goadsby PJ (2018). Cluster headache: epidemiology, pathophysiology, clinical features and diagnosis. Ann Indian Acad Neurol.

[8] Pascual J, Colás R, Castillo J (2001). Epidemiology of chronic daily headache. Curr Pain Headache Rep.

[9] Aaseth K, Grande RB, Kvárner KJ, Gulbrandsen P, Lundqvist C, Russell MB (2008). Prevalence of secondary chronic headaches in a population-based sample of 30-44-year-old persons. The Akershus study of chronic headache. Cephalalgia.

[10] Owolabi LF, Gwaram B (2012). Clinical profile of primary headache disorders in Kano, North western Nigeria. J Med Trop.

[11] Gidibidi GK, Kareemsab D, Rachaiah NM (2012). The socio-demographic profile, classification and the clinical profile of headache: a semi-urban hospital based study. J Clin Diagn Res.

[12] Delaruelle Z, Ivanova TA, Khan S (2018). Male and female sex hormones in primary headaches. J Headache Pain.

[13] Sacco S, Ricci S, Degan D, Carolei A (2012). Migraine in women: the role of hormones and their impact on vascular diseases. J Headache Pain.

[14] Singh S, Sarda K, Hegde R (2017). A Pan-India study to assess the quality of life, symptom profile and management trends in patients with migraine: a cross-sectional study. J Assoc Physicians India.

[15] Senthil C, Gunasekaran N (2016). Clinical profile of patients with chronic headache in a tertiary care hospital. Int J Adv Med.

[16] Chowdhury D (2012). Tension type headache. Ann Indian Acad Neurol.

[17] Tai MLS, Jivanadham JS, Tan CT, Sharma VK (2012). Primary headache in the elderly in South-East Asia. J Headache Pain.

[18] Xu H, Han W, Wang J, Li M (2016). Network meta-analysis of migraine disorder treatment by NSAIDs and triptans. J Headache Pain.

[19] Vatzaki E, Straus S, Dogne JM, Garcia Burgos J, Girard T, Martelletti P (2018). Latest clinical recommendations on valproate use for migraine prophylaxis in women of childbearing age: overview from European medicines agency and European headache federation. J Headache Pain.

